# Long non‐coding RNA SNHG6 promotes cell proliferation and migration through sponging miR‐4465 in ovarian clear cell carcinoma

**DOI:** 10.1111/jcmm.14359

**Published:** 2019-05-22

**Authors:** Yong Wu, Yu Deng, Qinhao Guo, Jun Zhu, Lijie Cao, Xueqi Guo, Fei Xu, Weiwei Weng, Xingzhu Ju, Xiaohua Wu

**Affiliations:** ^1^ Department of Gynecologic Oncology Fudan University Shanghai Cancer Center Shanghai People's Republic of China; ^2^ Department of Oncology Shanghai Medical College, Fudan University Shanghai People's Republic of China; ^3^ Cancer Institute, Fudan University Shanghai Cancer Center Shanghai People's Republic of China; ^4^ Department of Pathology Fudan University Shanghai Cancer Center Shanghai People's Republic of China

**Keywords:** cell migration, EZH2, miR‐4465, ovarian clear cell carcinoma, SNHG6

## Abstract

Dysregulation of small nucleolar RNA host gene 6 (SNHG6) exerts critical oncogenic effects and facilitates tumourigenesis in human cancers. However, little information about the expression pattern of SNHG6 in ovarian clear cell carcinoma (OCCC) is available, and the contributions of this long non‐coding RNA to the tumourigenesis and progression of OCCC are unclear. In the present study, we showed via quantitative real‐time PCR that SNHG6 expression was abnormally up‐regulated in OCCC tissues relative to that in unpaired normal ovarian tissues. High SNHG6 expression was correlated with vascular invasion, distant metastasis and poor survival. Further functional experiments demonstrated that knockdown of SNHG6 in OCCC cells inhibited cell proliferation, migration and invasion in vitro as well as tumour growth in vivo. Moreover, SNHG6 functioned as a competing endogenous RNA (ceRNA), effectively acting as a sponge for miR‐4465 and thereby modulating the expression of enhancer of zeste homolog 2 (EZH2). Taken together, our data suggest that SNHG6 is a novel molecule involved in OCCC progression and that targeting the ceRNA network involving SNHG6 may be a treatment strategy in OCCC.

## 1 INTRODUCTION

Epithelial ovarian cancer (EOC) is the most lethal gynaecological malignancy in the world, and its incidence has increased in the last decade.^1^, ^2^, ^3^ Of the several subtypes of EOC, ovarian clear cell carcinoma (OCCC) represents 5%‐25% of all EOCs depending on geographic location, and gene expression studies support the idea that OCCC is distinct from other EOCs, with a poorer prognosis due to a lower response rate to anticancer drug treatment.^3^, ^4^, ^5^ Identifying novel therapeutic targets and establishing new treatment strategies for OCCC is thus important.

In recent years, long non‐coding RNAs (lncRNAs) have been reported as a category of non‐coding RNAs with a length of greater than 200 nucleotides that do not encode proteins, and the emerging impact of lncRNAs in cancer initiation and progression has been discovered in diverse types of cancer, including OCCC.^6^, ^7^, ^8^ Accumulating evidence indicates that lncRNAs are involved in a variety of biological processes such as cell apoptosis, proliferation, metastasis, chemotherapeutic drug resistance and differentiation, suggesting that lncRNAs can be useful diagnostic markers or therapeutic agents for cancers.^6^, ^9^ For example, overexpression of MALAT1 and PVT1 promotes cancer cell proliferation and survival in gastrointestinal tumours.^10^, ^11^, ^12^ However, lncRNA‐p21 was reported to be down‐regulated and to suppress the growth and metastasis of cancer cells.^13^, ^14^

Currently, some small nucleolar RNAs (snoRNAs), a subclass of lncRNAs, exhibit differential expression patterns in various human cancers and demonstrate the ability to affect cell transformation, tumourigenesis and metastasis.^15^, ^16^ Small nucleolar RNA host gene 6 (SNHG6), also known as U87HG, is a recently identified lncRNA shown to be a potential oncogene involved in cell proliferation and epithelial‐mesenchymal transition (EMT) progression in many cancers.^17^, ^18^, ^19^, ^20^, ^21^, ^22^ However, the activities of SNHG6 related to OCCC tumourigenesis have not been well characterized, prompting us to explore the role of SNHG6 in human OCCC. In this study, we revealed that SNHG6 was overexpressed in OCCC tissues and that this lncRNA promoted cell proliferation, migration and invasion in vitro as well as tumour growth in vivo. Mechanistically, SNHG6 facilitated the development of OCCC by sponging miR‐4465, which targets enhancer of zeste homolog 2 (EZH2). In summary, our study revealed the role of SNHG6 and first revealed that SNHG6 could sponge miR‐4465 in OCCC.

## 2 MATERIALS AND METHODS

### 2.1 Human samples and tissue handling

This study was conducted with the understanding and written consent of each individual. The study methodologies conformed to the standards established by the Declaration of Helsinki. All human tissues were collected using the protocols approved by the Human Ethics Committee of the Fudan University Shanghai Cancer Center. Ovarian clear cell carcinoma tissues were obtained from 48 patients who underwent surgical resection of ovarian cancer between 2012 and 2014. Forty‐four normal ovarian tissues were collected from patients undergoing cervical cancer surgery. No local or systemic treatment was administered to these patients prior to the operation. All tissue samples were washed with sterile PBS before being snap‐frozen in liquid nitrogen and stored at −80°C until analysis. The pathological parameters were evaluated by an experienced pathologist. Patient follow‐up was performed every 3 months during the first post‐surgical year and every 6 months thereafter until 1 January 2018. Disease‐free survival (DFS) was calculated from the date of surgery to the date of recurrence or final follow‐up as appropriate.

### 2.2 Cell lines and culture conditions

Cells were obtained as follows: the HEK‐293T and ES‐2 cell lines were purchased from the Cell Bank of Type Culture Collection (CBTCC, Chinese Academy of Sciences, Shanghai, China). The RMG‐1, TOV21G, OVCA420 and OVISE cell lines were purchased from Jingdu Biotech (Shanghai, China). All six cell lines were cultured in DMEM (HyClone). All media were supplemented with 10% foetal bovine serum (10% FBS, Gibco), 100 U/mL penicillin (Gibco) and 100 mg/mL streptomycin (Gibco), and all cell lines were maintained at 37°C in a humidified atmosphere of 5% CO_2_.

### 2.3 RNA isolation, reverse transcription and quantitative real‐time PCR

As described in previous reports, total RNA was extracted from the tissue samples and cell lines using TRIzol reagent (Invitrogen, Carlsbad, CA) according to the manufacturer's protocol. Reverse transcription (RT) and quantitative real‐time PCR (qRT‐PCR) kits (Takara, Dalian, China) were utilized to evaluate the mRNA expression levels of the indicated genes. PCR primers were designed and synthesized using a primer design tool (Vector NTI; The primers used in this study are listed in Table S1). The relative quantification value for each target gene was expressed as 2^−ΔΔCT^. β‐Actin was used as the internal reference for the mRNA expression, and U6 was used as the internal reference for miRNA expression.

### 2.4 Plasmids, lentivirus construction, small interfering RNAs and cell transfections

Small nucleolar RNA host gene 6 was cloned into the expression vector pcDNA3.1 for overexpression (Lingke Biotech). SNHG6 short hairpin RNA (shRNA) lentivirus for use in the in vivo experiments was purchased from ABM Company. Small interfering RNAs (siRNAs) targeting SNHG6 were purchased from Suzhou Synbio Technologies and transfected into cells using Lipofectamine 3000 (Invitrogen). All siRNA and shRNA sequences are provided in Table S2. miR‐4465 mimics were purchased from Synbio Technologies (Suzhou, China).

### 2.5 Cell proliferation assays

A total of 2 × 10^3^ cells per well were seeded in 96‐well plates 24 hours before the experiment. TOV21G and RMG‐1 cells were transfected with targeted siRNAs or scrambled siRNA. Proliferation was measured using a Cell Counting Kit‐8 (CCK‐8) kit (Dojindo, Japan) according to the manufacturer's protocol. All experiments were performed in triplicate. Cell proliferation curves were plotted using the absorbance at each time point.

### 2.6 Colony formation assay

Cells were digested with trypsin into single‐cell suspensions at 48 hours after transfection. For the colony formation assay, a sample of 2000 cells was plated into six‐well plates and incubated in the appropriate medium supplemented with 10% FBS at 37°C. After 2 weeks, cells were fixed and stained with 0.1% crystal violet, and visible colonies were manually counted. Triplicate wells were measured for each treatment group.

### 2.7 Cell wound healing and invasion assays

For the wound healing assay, cells were seeded into six‐well plates and allowed to grow to 90%‐95% confluence. A single scratch wound was created 6 hours after siRNA transfection. Cells were washed with PBS to remove cell debris, supplemented with serum‐free medium and monitored. Images were captured by phase contrast microscopy at 0, 24, 36 and 48 hours after wounding.

A cell invasion assay was performed with Transwell chamber inserts (8.0 mm, Corning, NY) in a 24‐well plate. A total of 4 × 10^4^ cells suspended in 200 µL of serum‐free medium were added to the upper chamber. Culture medium containing 20% FBS was placed in the bottom chamber. Cells were incubated for 36 or 48 hours at 37°C. After incubation, cells on the upper surface were removed by scraping and washing, whereas cells on the lower surface were fixed with 20% methanol and stained with 0.1% crystal violet. The number of invaded cells in five randomly selected fields was counted under a microscope. The experiments were repeated independently in triplicate.

### 2.8 Western blotting

Cells were lysed in RIPA buffer (Sigma‐Aldrich) supplemented with protease inhibitor (Roche, Basel, Switzerland) and phosphatase inhibitor (Roche). The protein concentration was measured using a BCA protein assay kit (Thermo Scientific, USA). Rabbit anti‐EZH2, anti‐MMP2, anti‐MMP9, anti‐N‐Cadherin and anti‐E‐Cadherin primary antibodies were purchased from Cell Signaling Technology.

### 2.9 In vivo experiments and immunohistochemical staining

Female BALB/c nude mice 6‐8 weeks of age were purchased from the Shanghai Laboratory Animal Center at the Chinese Academy of Sciences. All experiments were performed in accordance with relevant institutional and national guidelines and the regulations of the Shanghai Medical Experimental Animal Care Commission. Mice (five per group) were injected subcutaneously with 0.2 mL of a cell suspension containing 8 × 10^6^ cells (the piLenti‐shRNA‐VECTOR and piLenti‐shRNA‐SNHG6 stable TOV21G cell lines) in the right axilla. The tumour growth rates were monitored. Palpable tumours were measured every other day, and tumour volume was calculated according to the following formula: volume = length × width^2^ × 0.5. Mice were killed 6 weeks after injection, and the tumour weights were recorded.

Immunohistochemical staining was then performed. Specimens were incubated with anti‐Ki‐67 antibody (1:100; Servicebio, China) overnight at 4°C and were then incubated with a biotinylated secondary antibody (1:100, goat anti‐rabbit/IgG) for 30 minutes at 37°C.

### 2.10 Subcellular fractionation

Separation of the nuclear and cytosolic fractions of TOV21G and RMG‐1 cells was performed with a PARIS kit (Life Technologies; Thermo Fisher Scientific, Inc), according to the manufacturer's protocol.

### 2.11 Luciferase assay

The wild‐type (WT) or mutant (MUT) SNHG6 binding sites were subcloned into the pGL3‐Basic vector (Promega, Madison, WI). HEK‐293T cells were seeded into 96‐well plates. miR‐4465 mimics or a negative control (NC) sequence (RiboBio, Guangzhou, China) were cotransfected with pGL3‐SNHG6 or pGL3‐SNHG6‐MUT. Two days after transfection, cells were collected, and luciferase activity was determined using a Dual Luciferase Reporter Assay System (Yeason, Shanghai, China). Similarly, the EZH2‐3′UTR and EZH2‐3′UTR‐MUT plasmids containing the putative binding site of miR‐4465 were constructed and cloned into a firefly luciferase expression vector (RiboBio). HEK‐293T cells were seeded into 96‐well plates and transfected with either pGL3‐EZH2‐3′UTR or pGL3‐EZH2‐3′UTR‐MUT reporter vector, together with a miR‐106b‐5p mimic or NC using Lipofectamine 3000 (Invitrogen). After 48 hours, cells were harvested, and the firefly and Renilla luciferase activities were measured using the dual luciferase reporter assay system (Yeason).

### 2.12 Statistical analysis

All statistical analyses were performed with spss 18.0 (IBM, SPSS, Chicago, IL). The significance of the differences between the groups was estimated using Student's *t* test, the chi‐squared test or the Wilcoxon test as appropriate. The overall survival (OS) and DFS rates were calculated using the Kaplan‐Meier method with the log‐rank test for comparison. A value of *P* < 0.05 indicated a significant difference.

## 3 RESULTS

### 3.1 SNHG6 is up‐regulated in OCCC and is associated with poor prognosis

**Figure 1:**
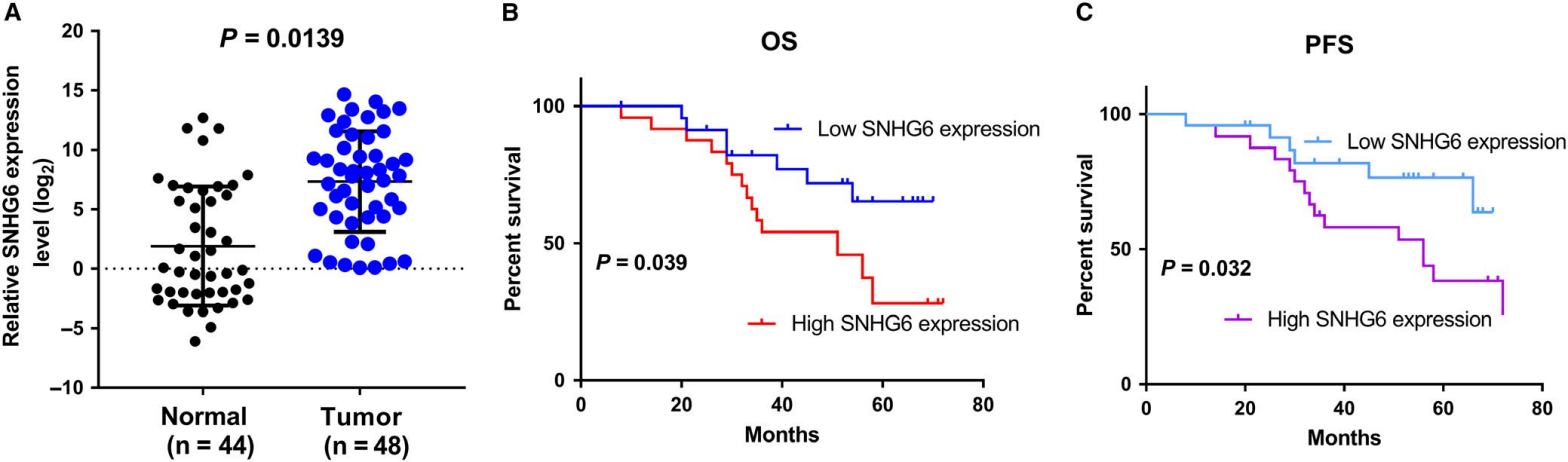
Small nucleolar RNA host gene 6 (SNHG6) is up‐regulated in ovarian clear cell carcinoma (OCCC) and is correlated with patient prognosis. A, Small nucleolar RNA host gene 6 expression levels in OCCC and unpaired normal ovarian tissues. B, Kaplan‐Meier analysis of overall survival (OS) in all patients with OCCC according to SNHG6 expression. C, Kaplan‐Meier curves for the progression‐free survival (PFS) of patients based on SNHG6 expression

To explore the role of SNHG6 in OCCC, we first examined its expression in 48 patients with OCCC using qRT‐PCR. The results showed higher SNHG6 expression in OCCC tissues than in normal ovarian tissues (*P* = 0.0139; Figure 1A). In addition, clinicopathological correlation analysis was conducted, and the 48 OCCC patients were divided into the high‐ and low‐expression groups based on the median expression value. As shown in Table 1, high‐SNHG6 expression was strongly associated with vascular invasion and distant metastasis, suggesting that SNHG6 may be involved in OCCC invasion and metastasis. The OS and progression‐free survival (PFS) curves were then plotted according to the SNHG6 expression level using the Kaplan‐Meier method. As shown in Figure 1B and 1, high‐SNHG6 expression was significantly correlated with both shorter OS (*P* = 0.039) and shorter PFS (*P* = 0.032) times.

**Table 1:** The correlation between SNHG6 and clinicopathological parameters

Variables	n (%)	Expression of SNHG6		*χ* ^2^	*P* value
		Low	High		
Age (y)				0.000	0.614
≤55	22 (45.8)	11	11		
>55	26 (54.2)	13	13		
Size (cm)				0.403	0.376
≤5	34 (70.8)	18	16		
>5	14 (29.2)	6	8		
FIGO stage				0.105	0.500
I‐II	13 (27.1)	7	6		
III–IV	35 (72.9)	17	18		
Poor histologic differentiation				0.336	0.386
Yes	22 (45.8)	10	12		
No	26 (54.2)	14	12		
Vascular invasion				6.762	0.010*
Yes	23 (47.9)	7	16		
No	25 (52.1)	17	8		
Lymphatic metastasis				0.085	0.500
Yes	21 (43.8)	8	10		
No	27 (56.2)	16	11		
Distant metastasis				4.148	0.040*
Yes	21 (43.8)	7	14		
No	27 (52.1)	17	10		

Abbreviations: FIGO, International Federation of Gynecology and Obstetrics; SNHG6, small nucleolar RNA host gene 6. *P < 0.05.

### 3.2 SNHG6 promotes the proliferation of OCCC cells

**Figure 2:**
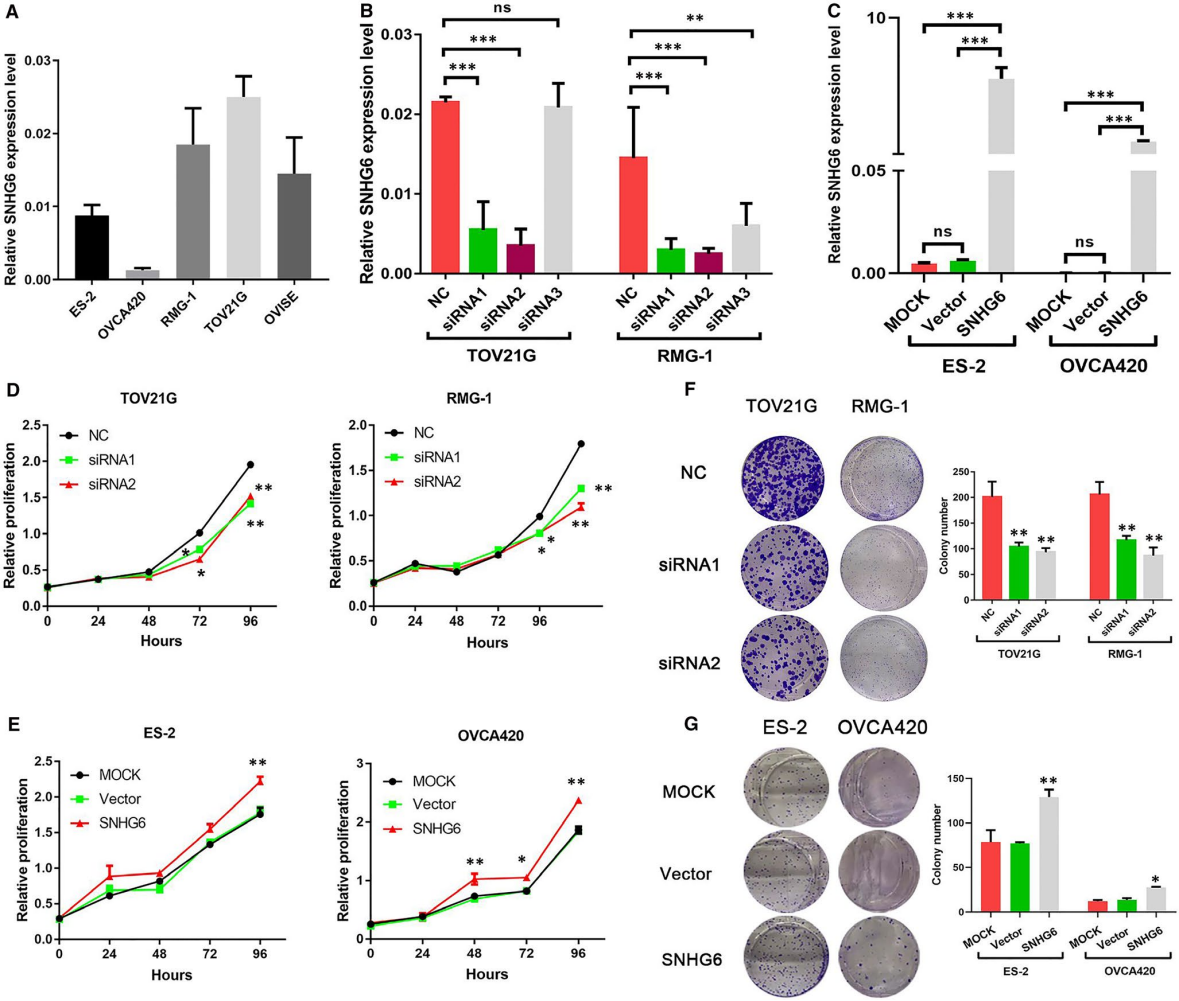
Small nucleolar RNA host gene 6 (SNHG6) promotes cell proliferation in vitro. **A**, Small nucleolar RNA host gene 6 expression was quantitated in five ovarian clear cell carcinoma (OCCC) cell lines using quantitative real‐time PCR (qRT‐PCR). (B,C) The OCCC cell lines TOV21G and RMG‐1 were transfected with small interfering RNAs (siRNAs), and the ES‐2 and OVCA420 cell lines were transfected with SNHG6 overexpression plasmids. The efficiency of knockdown or overexpression was verified by qRT‐PCR. (D,E) Cell Counting Kit‐8 assays were performed to determine the influence of SNHG6 on cell proliferation. (F,G) The cell colony‐forming ability of OCCC cells was assessed to determine the effects of SNHG6 on cell growth. *P < 0.05, **P < 0.01, ***P < 0.001 vs negative control (NC). ns, not significant

Aiming to identify a function of SNHG6, we profiled its expression in a panel of OCCC cell lines. SNHG6 expression was then down‐ or up‐regulated in TOV21G, RMG‐1, ES‐2 and OVCA420 cell lines in accordance with their high‐ or low‐endogenous SNHG6 expression (Figure 2A). Transfection with SNHG6 siRNAs or the overexpression plasmid significantly down‐ or up‐regulated the expression of SNHG6 respectively (Figure 2B and 2). As demonstrated by the CCK‐8 assays, two of the three independent siRNAs targeting SNHG6 significantly decreased the proliferation of TOV21G and RMG‐1 cells (Figure 2D), whereas the overexpression of SNHG6 led to a significant increase in the growth of ES‐2 and OVCA420 cells (Figure 2E). Consistent with these results, repression of SNHG6 significantly inhibited the colony‐forming ability of OCCC cells (Figure 2F), whereas SNHG6 overexpression accelerated OCCC cell colony formation compared with that in the control groups (Figure 2G).

### 3.3 SNHG6 enhances the invasion and migration of OCCC cells

**Figure 3:**
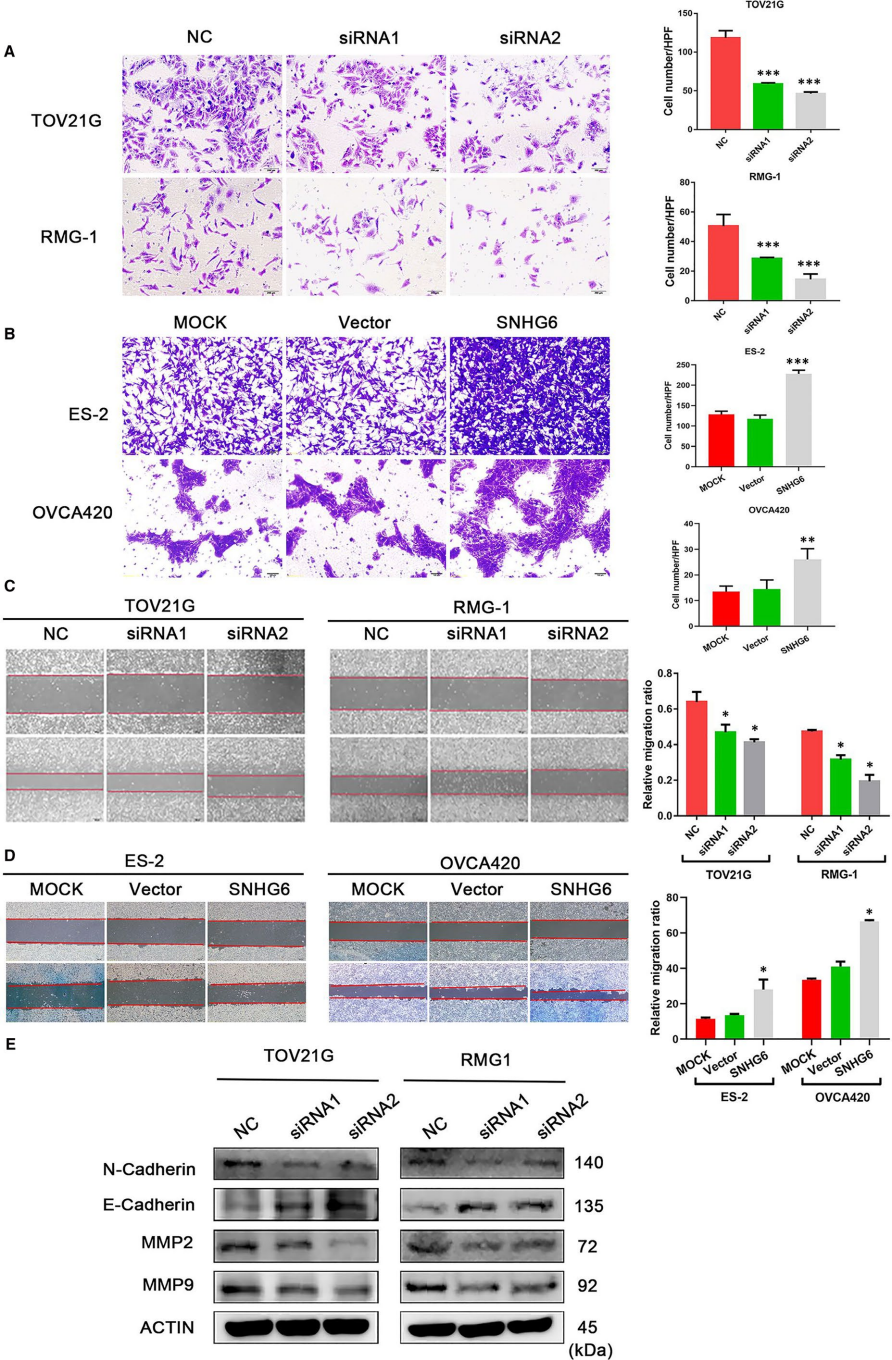
Small nucleolar RNA host gene 6 (SNHG6) enhances cell invasion and migration in vitro. (A,B) The invasion potential of cells transfected with the SNHG6 small interfering RNAs (siRNAs) or the overexpression plasmid was assessed using a Transwell assay. The scale bar represents 50 μm. (C,D) The migration ability of cells with altered SNHG6 expression was evaluated using a wound healing assay; images of TOV21G, RMG‐1, ES‐2 and OVCA420 cells were captured at 0 and 48 h post wounding. The scale bar represents 200 μm. E, Western blot analysis was used to determine the N‐cadherin, E‐Cadherin, MMP2 and MMP9 expression levels. β‐Actin or cytoplasmic 1 (ACTIN) was used as the reference. *P < 0.05, **P < 0.01, ***P < 0.001 vs negative control (NC)

To determine whether SNHG6 promotes OCCC cell invasion and migration, we performed Transwell and wound healing assays. Knockdown of SNHG6 resulted in a decrease in the number of invaded cells relative to the number of invaded control cells (*P* < 0.05; Figure 3A); conversely, SNHG6 induction increased the invasive ability of ES‐2 and OVCA420 cells (*P* < 0.05; Figure 3B). Moreover, the migration capacity was decreased when SNHG6 was down‐regulated (*P* < 0.05; Figure 3C). The migration assay results were verified by pcDNA3.1‐SNHG6 transfection (*P* < 0.05; Figure 3D). Accordingly, the Western blotting results showed that SNHG6 knockdown down‐regulated the expression of invasion‐promoting proteins such as N‐cadherin, MMP2 and MMP9 and up‐regulated the expression of the invasion‐suppressing protein E‐cadherin (Figure 3E). Collectively, these results suggest that SNHG6 may enhance OCCC cell migration and invasion.

### 3.4 SNHG6 knockdown inhibits the growth of OCCC xenografts in vivo

**Figure 4:**
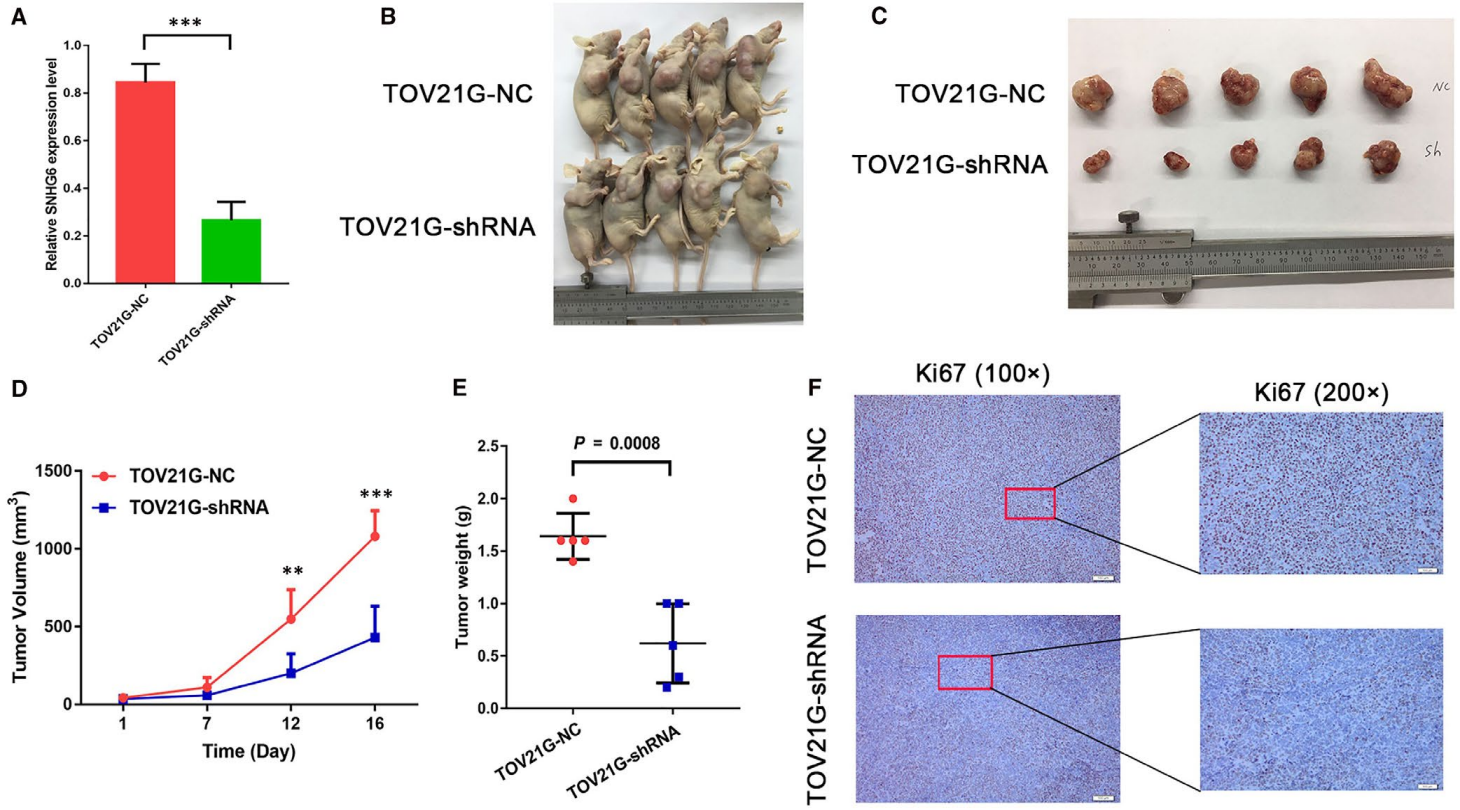
Knockdown of small nucleolar RNA host gene 6 (SNHG6) inhibits ovarian clear cell carcinoma (OCCC) growth in vivo. A, Cell lines with stable SNHG6 knockdown were constructed, and the knockdown efficiency was assessed by quantitative real‐time PCR (qRT‐PCR). (B,C) Down‐regulation of SNHG6 expression attenuated tumour growth in nude mice. (D,E) The effect of SNHG6 on OCCC tumour growth was evaluated based on the tumour volumes and tumour weights in the two groups. F, An immunohistochemical staining assay was conducted to compare the proliferation indexes via Ki‐67 expression levels. The scale bars represent 50 and 200 μm. **P < 0.01, ***P < 0.001 vs negative control (NC)

To provide in vivo evidence for the oncogenic role of SNHG6 in OCCC, we established cell lines with stable SNHG6 knockdown for use in a xenograft mouse model; the knockdown efficiency is shown in Figure 4A. Ten mice were injected subcutaneously with TOV21G‐NC and TOV21G‐shRNA cells, and all developed detectable tumours (Figure 4B). However, knockdown of SNHG6 markedly reduced the increase in the tumour volumes in the xenograft mouse model (Figure 4C). Additionally, the size and weight of SNHG6 knockdown tumours were lower than those of NC tumours (Figure 4D and 4). In addition, Ki‐67 staining, which represents the proliferation index, was lower in the SNHG6 knockdown groups than in the control groups (Figure 4F). Therefore, SNHG6 may promote OCCC tumourigenesis both in vitro and in vivo.

### 3.5 SNHG6 functions as a sponge for miR‐4465 in OCCC cells

**Figure 5:**
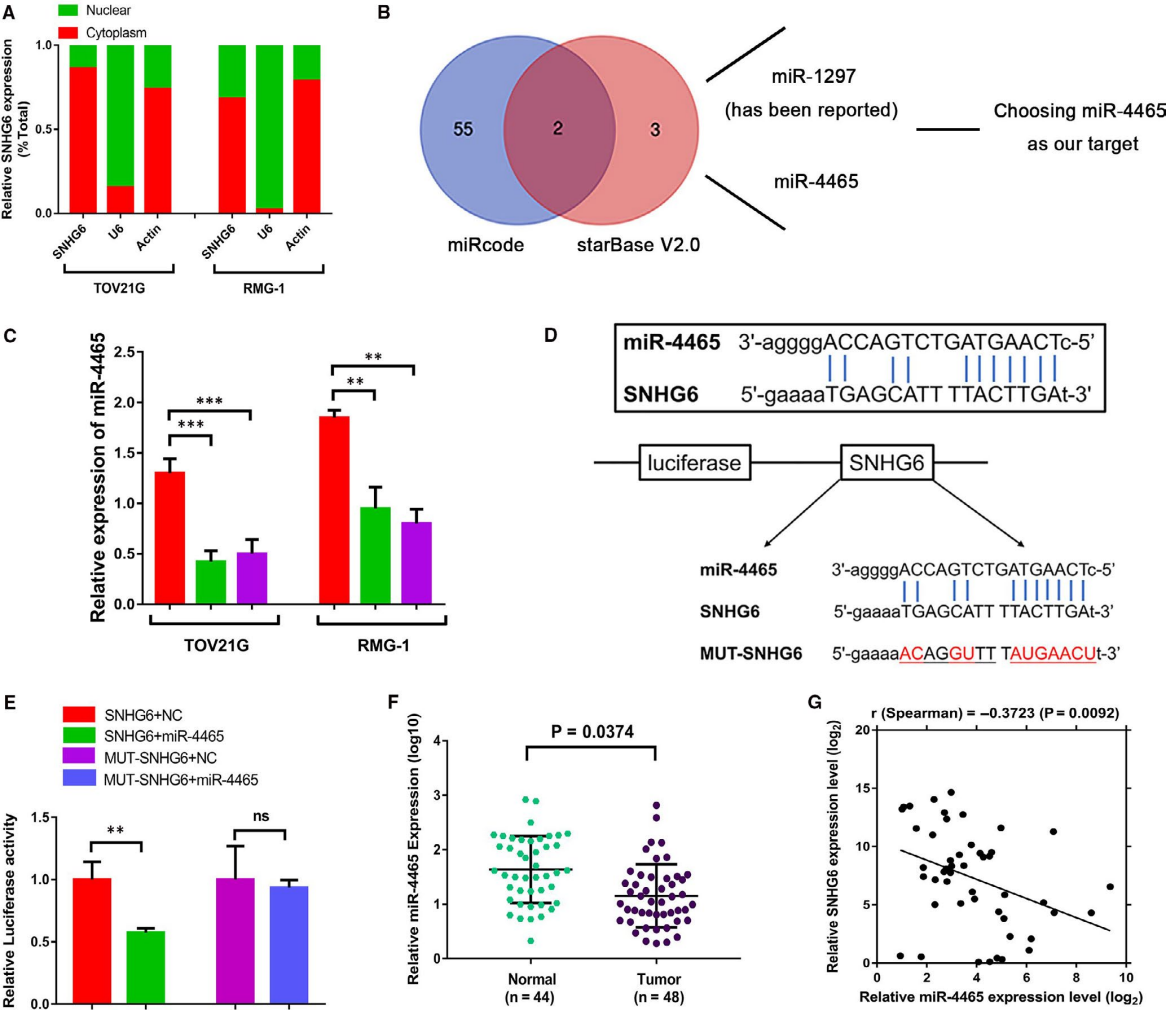
Small nucleolar RNA host gene 6 (SNHG6) functions as a sponge for miR‐4465. A, The cytoplasmic and nuclear RNA fractions were isolated from TOV21G and RMG‐1 cells. Small nucleolar RNA host gene 6 was distributed mainly in the cytoplasm. β‐Actin was the cytoplasmic internal control, and U6 was the nuclear internal control. The values are presented as the means ± SEMs. B, Venn diagram shows the results of the combination analysis to identify potential targets of SNHG6 (miRcode: http://www.mircode.org/; starBase V2.0: http://starbase.sysu.edu.cn/mirLncRNA.php). C, The relative miR‐4465 levels in TOV21G and RMG‐1 cells with SNHG6 knockdown are shown. D, The binding region between miR‐4465 and SNHG6 was predicted, and diagrams of the luciferase reporter plasmids containing the wild‐type (WT) (SNHG6) or mutant SNHG6 (MUT‐SNHG6) sequence are shown. E, Small nucleolar RNA host gene 6 cDNA containing the putative miR‐4465 binding site or the corresponding mutant sequence was cloned downstream of the luciferase gene in the pGL3‐Basic vector; the resulting plasmid was designated RLuc‐SNHG6. Luciferase reporter plasmids containing the WT or mutant SNHG6 sequence were then cotransfected into HEK‐293T cells along with miR‐4465 mimics in parallel with an empty plasmid vector. Luciferase activity was determined using a dual luciferase assay and is shown as the relative luciferase activity normalized to Renilla luciferase activity. F, The differential expression of miR‐4465 in ovarian clear cell carcinoma (OCCC) tissues (n = 48) and unpaired normal ovarian tissues (n = 44) was analysed. G, Pearson correlation curves are shown, revealing the negative relationship between SNHG6 and miR‐4465 in OCCC. **P < 0.01, ***P < 0.001 vs negative control (NC). ns, not significant

To understand the mechanism by which SNHG6 contributes to the malignant phenotypes of OCCC cells, we assessed SNHG6 localization, because the activities of lncRNAs mainly depend on their subcellular distribution. Analysis of the cytoplasmic and nuclear RNA fractions of OCCC cells revealed that SNHG6 was localized preferentially in the cytoplasm (Figure 5A). Previous literature reported that cytoplasmic lncRNAs can bind directly to miRNAs and function as sponges or as competing endogenous RNAs (ceRNAs) to control the availability of miRNAs for binding to their target mRNAs. Bioinformatics analysis using miRcode (http://www.mircode.org/) and starBase v2.0 (http://starbase.sysu.edu.cn/mirLncRNA.php) software predicted that SNHG6 could bind miR‐4465 and miR‐1297; miR‐4465 binding had not been reported previously (Figure 5B). The prediction of binding sites between SNHG6 and miRNAs are listed in Tables S3 and S4. In addition, the qRT‐PCR results showed that the expression of miR‐4465 was up‐regulated after SNHG6 knockdown (Figure 5C); thus, miR‐4465 was selected as the predicted candidate. To validate whether miR‐4465 was a direct target of SNHG6, luciferase reporter plasmids expressing SNHG6 with WT/MUT miR‐4465 binding sites were constructed (Figure 5D). Cotransfection of HEK‐293T cells with the luciferase reporter plasmid containing the WT binding sites and miR‐4465 mimics decreased the reporter activity relative to that in NC cells (Figure 5E). Considering the interaction between SNHG6 and miR‐4465 in OCCC cells, we examined the miR‐4465 levels in OCCC patients. The qRT‐PCR results revealed that the miR‐4465 level was much higher in normal ovarian tissues than in OCCC tissues (Figure 5F). Moreover, Spearman correlation analysis suggested a significant negative correlation between the expression levels of SNHG6 and miR‐4465 in OCCC tissues (*r *= −0.3723, *P* = 0.0092; Figure 5G), further confirming the relationship between SNHG6 and miR‐4465.

### 3.6 SNHG6 influences the expression of the miR‐4465 target gene EZH2

**Figure 6:**
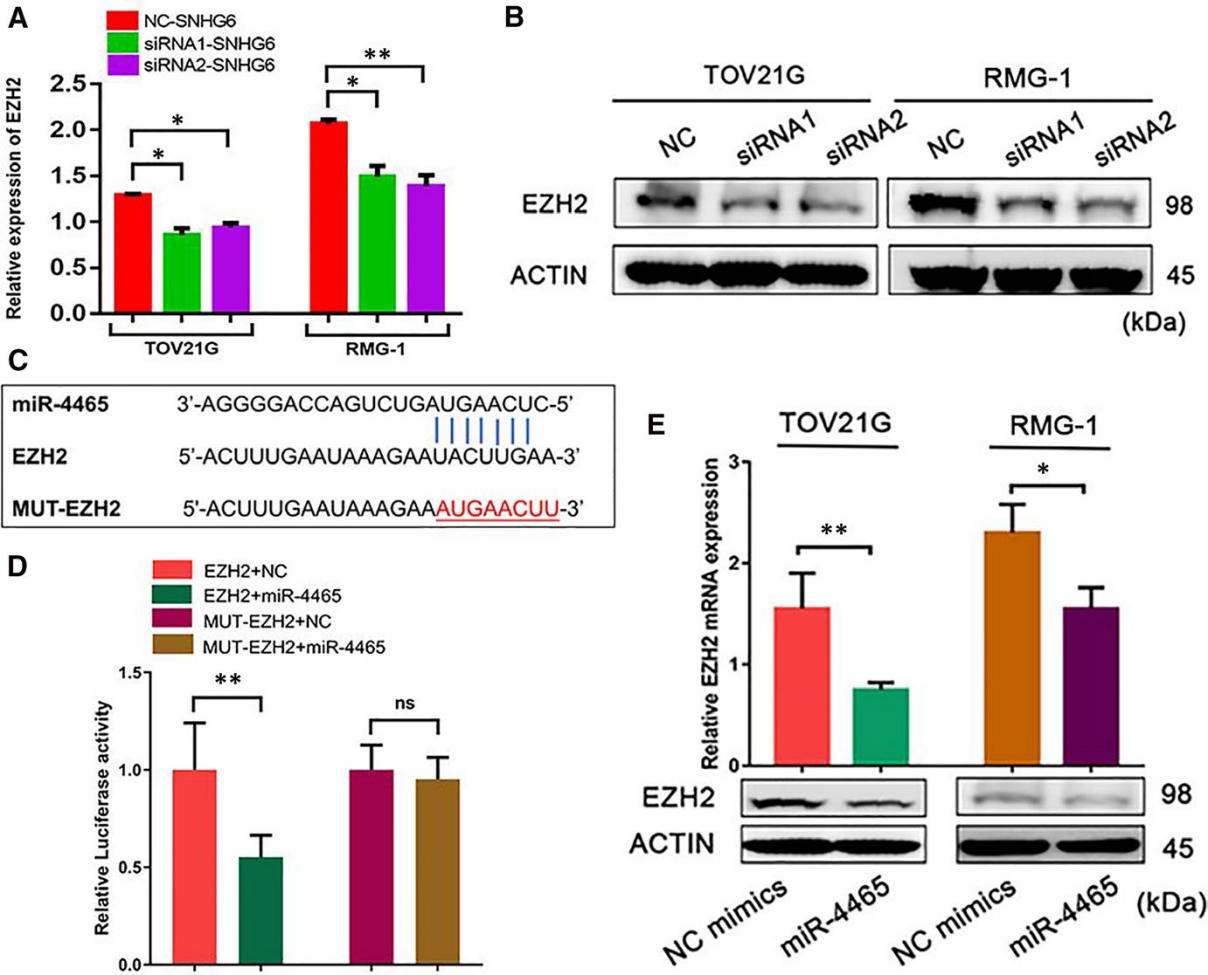
The small nucleolar RNA host gene 6 (SNHG6)/miR‐4465 axis promotes ovarian clear cell carcinoma (OCCC) development by influencing enhancer of zeste homolog 2 (EZH2) expression. A, Knockdown of SNHG6 influenced EZH2 mRNA levels. B, Knockdown of SNHG6 influenced EZH2 protein levels. C, The binding region between miR‐4465 and EZH2 was identified via bioinformatic database (TargetScan) analysis and a literature review. D, Vector‐EZH2/MUT‐EZH2 was cotransfected into HEK‐293T cells with miR‐4465 mimics. Luciferase activity was measured by a dual luciferase assay. The data are presented as the relative luciferase activity normalized to the Renilla luciferase activity. E, mRNA and protein expression of EZH2 in OCCC cells after 48 h of transfection with miR‐4465 mimics. *P < 0.05, **P < 0.01 vs negative control (NC)

To identify the targets of SNHG6 ceRNA, target prediction tools (TargetScan; http://www.targetscan.org/vert_50) were used and a literature review was conducted to evaluate the potential miR‐4465 target genes. Among these potential target genes, EZH2 was selected as the predicted target. Enhancer of zeste homolog 2 is also considered an important factor in EMT, which is associated with tumour growth and metastasis. We first examined whether SNHG6 could influence EZH2 expression and found that depletion of SNHG6 down‐regulated EZH2 mRNA and protein expression in TOV21G and RMG‐1 cells (Figure 6A and 6), suggesting that SNHG6 influences EZH2 by sponging miR‐4465. To further assess whether EZH2 was a direct target of miR‐4465, luciferase reporter plasmids containing the WT and MUT EZH2 binding sites were designed (Figure 6C). Cotransfection of the luciferase reporter plasmid containing the WT EZH2 binding site with miR‐4465 mimics into HEK‐293T cells contributed to a decrease in reporter activity (Figure 6D). In addition, EZH2 expression was decreased by miR‐4465 mimics in TOV21G and RMG‐1 cells (Figure 6E). These results suggest that SNHG6 promotes OCCC cell proliferation and invasion through reducing miR‐4465 binding to EZH2 mRNA.

### 3.7 SNHG6 facilitates tumour proliferation and metastasis via miR‐4465

**Figure 7:**
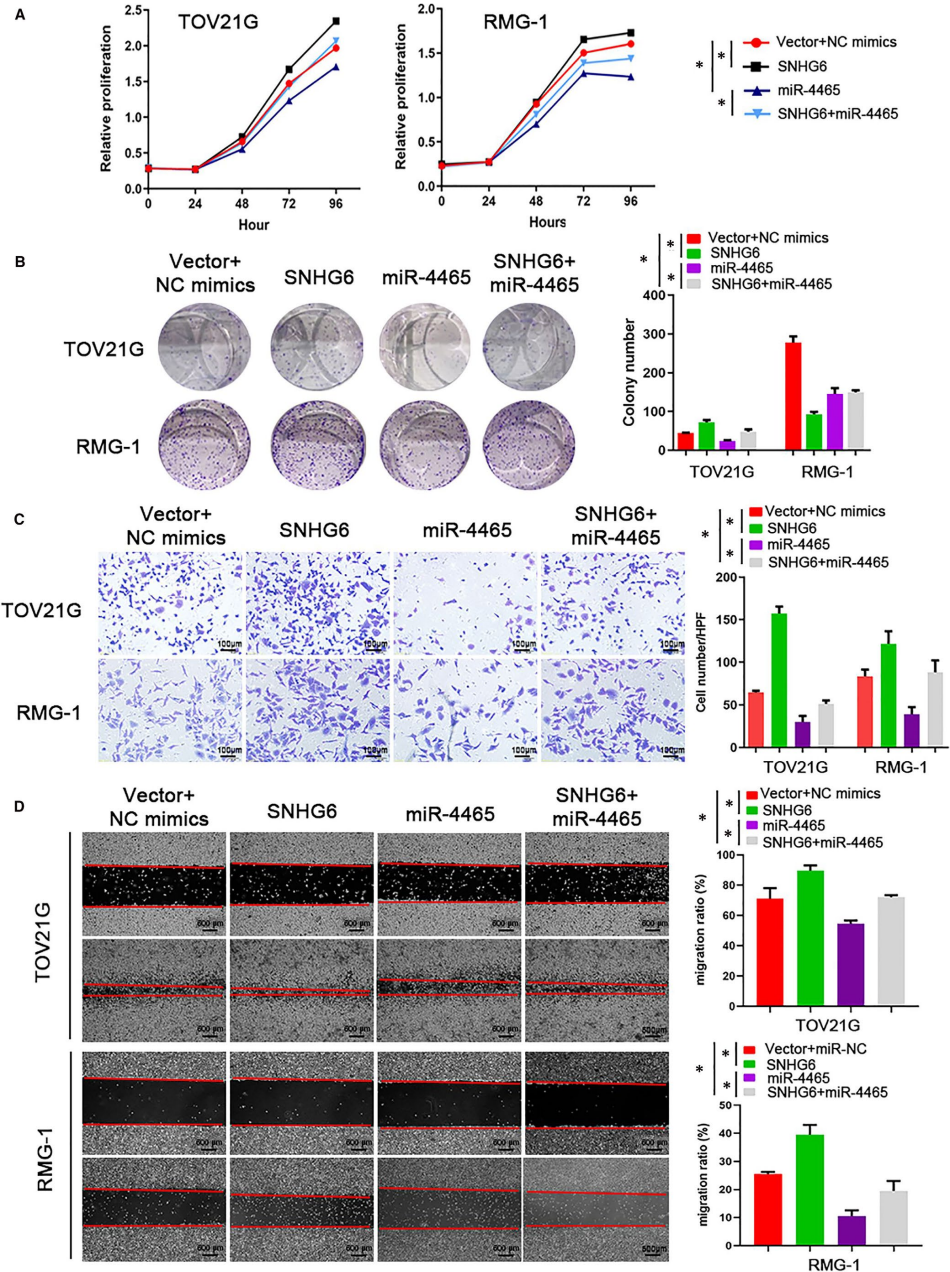
Small nucleolar RNA host gene 6 (SNHG6) exerts pro‐tumour effects by inhibiting miR‐4465. A, Growth curves of TOV21G and RMG‐1 cells cotransfected with SNHG6, miR‐4465 mimics or control constructs were determined via Cell Counting Kit‐8 assays. B, Colony formation assays were performed to detect the proliferative ability of the indicated ovarian clear cell carcinoma cells. C, Transwell invasion rescue experiments were performed after cotransfection of SNHG6‐overexpressing cells with miR‐4465 mimics. D, Representative images of the wound healing assay of TOV21G and RMG‐1 cells with ectopic expression of SNHG6 and/or cotransfected with miR‐4465 mimics. Quantification data are shown in the histogram on the right. *P < 0.05 vs negative control (NC)

As we found that SNHG6 directly binds to miR‐4465, we next investigated the coregulation of OCCC cell proliferation and metastasis by SNHG6 and miR‐4465. The CCK‐8 and colony formation assay results showed that the proliferation of TOV21G and RMG‐1 cells was increased when SNHG6 expression was up‐regulated and inhibited when miR‐4465 was overexpressed; however, the promotive effect of the SNHG6 overexpression plasmid on OCCC cell growth was partially restored by transfection with miR‐4465 mimics (*P* < 0.05; Figure 7A and 7). In addition, the Transwell assay results showed that cell invasion was inhibited after transfection with miR‐4465 mimics, suggesting the suppressive effect of miR‐4465 on tumour invasion. As expected, the pro‐metastatic effect of SNHG6 in TOV21G and RMG‐1 cells was rescued by cotransfection with miR‐4465 mimics (*P* < 0.05; Figure 7C). Similarly, the SNHG6‐induced promotion of migration was reversed by miR‐4465 mimics (*P* < 0.05; Figure 7D).

## 4 DISCUSSION

Recently, the vast majority of lncRNA transcripts have been discovered through high‐throughput techniques.^23^, ^24^, ^25^ However, although these techniques quantify these transcripts, only a few have been shown to be involved in regulating a diverse array of biological processes.^26^ This study demonstrated the roles of SNHG6, whose functions in other cancers have been suggested in previous studies.^17^, ^18^, ^19^, ^20^ For example, Chang et al reported that SNHG6 promotes tumour growth and metastasis by inducing EMT in hepatocellular carcinoma.^27^ Guo et al showed the role of lncRNA SNHG6 in suppressing the production of the universal methyl donor SAMe and the impact of SAMe on global DNA methylation levels in liver cancer, also highlighting the potential benefit of SAMe for the treatment of liver cancer.^22^ In addition, SNHG6 was found to be up‐regulated in gastric cancer and to promote cell proliferation and EMT through epigenetically silencing p27 and sponging miR‐101‐3p.^20^ Although the biological function of SNHG6 has been explored in some types of cancer, the detailed mechanism by which SNHG6 acts in OCCC tumourigenesis requires further investigation. In the present study, we demonstrated that SNHG6 was overexpressed in OCCC tissues and is potentially correlated with OCCC prognosis. Moreover, this lncRNA promoted cell proliferation, migration and invasion in vitro as well as tumour growth in vivo. These results indicate that SNHG6 acts as an oncogene in OCCC and can be considered a potential prognostic indicator.

Emerging evidence suggests that lncRNAs might function as ceRNAs or as molecular sponges to modulate the activity of miRNAs.^28^, ^29^, ^30^ However, it is reported that not every lncRNA drives tumourigenesis by interacting with miRNAs; typically, cytoplasmic lncRNAs can sponge miRNAs.^30^, ^31^ Thus, to investigate the potential mechanism of SNHG6 in OCCC, subcellular fractionation was first conducted to assess SNHG6 localization. SNHG6 was mainly distributed in the cytoplasm, suggesting that it may function as an endogenous decoy for miRNAs. Subsequently, analysis of two databases showed that miR‐4465 was a target of SNHG6. Luciferase assays and Pearson correlation analysis further indicated that SNHG6 and miR‐4465 interact, a possible mechanism by which SNHG6 functions as an oncogene. In addition, we showed that the level of miR‐4465 was significantly decreased in OCCC tissues, consistent with the results of a previous study in lung cancer.^32^

Furthermore, we attempted to identify the downstream target of miR‐4465 in OCCC cells that may mediate the activity of the SNHG6/miR‐4465 axis. Then, we combined bioinformatics tools with previous studies for a comprehensive analysis. According to the bioinformatics database, EZH2, a critical oncogene that regulates multiple cellular processes in cancers, was a candidate target of miR‐4465. Indeed, previous studies confirmed that EZH2 is a target of miR‐4465 in cancer cells.^32^ The luciferase reporter assay results indicated that miR‐4465 directly targeted the EZH2 3’UTR. In addition, the qRT‐PCR and Western blotting results showed that miR‐4465 negatively regulated the mRNA and protein expression of EZH2. Based on our work, we propose a ceRNA model including SNHG6, miR‐4465 and EZH2 in OCCC. Previous studies have demonstrated that EZH2 plays a critical role in cell proliferation, cell invasion, tumour metastasis, angiogenesis and chemotherapy resistance in cancer.^33^, ^34^, ^35^ In our in vitro systems, ectopic expression of SNHG6 was sufficient to increase the expression of EZH2. Moreover, we confirmed that SNHG6 promoted OCCC cell proliferation and metastasis, which was restored by miR‐4465 mimics. These data suggest that SNHG6 exerts its pro‐tumour effects at least in part by regulating miR‐4465 expression.

In conclusion, this study is the first to investigate a potential mechanism of the SNHG6/miR‐4465 axis in OCCC progression. Increased expression levels of SNHG6 are associated with tumour progression and are inversely correlated with prognosis. In addition, SNHG6 functions as a ceRNA, regulating EZH2 expression by competitively binding miR‐4465. These findings provide mechanistic insight into the role of SNHG6 in promoting OCCC metastasis and suggest that SNHG6 is an important prognostic factor and therapeutic target.

## CONFLICT OF INTEREST

The authors declare that they have no conflict of interest.

## AUTHORS CONTRIBUTIONS

Yong Wu, Yu Deng and Xiaohua Wu designed the study and wrote the manuscript. Yong Wu, Yu Deng and Qinhao Guo performed all the experiments. Jun Zhu performed the statistical analyses. Lijie Cao, Xueqi Guo, Fei Xu and Xingzhu Ju provided valuable guidance in the experimental process. All the authors read and approved the final manuscript.
